# How do Professionals in Municipal Health and Welfare Relate to Bereaved Persons During the Acute Phase of a Drug-Related Death? A Qualitative Study

**DOI:** 10.1177/23333936221085035

**Published:** 2022-04-12

**Authors:** Hilde-Margit Løseth, Lillian B. Selseng, Kari Dyregrov, Sonja Mellingen

**Affiliations:** 1 1657 Western Norway University of Applied Sciences, Bergen, Norway

**Keywords:** drug-related death, bereaved, psychosocial follow-up, municipal first responder services, Norway, narkotikarelaterte dødsfall, etterlatte, narkotikarelatert psykososial oppfølging, kommunalt akuttberedskap, Norge

## Abstract

This study aims to broaden our knowledge of how professionals in municipal health and welfare relate to bereaved persons during the acute phase of a drug-related death. A reflexive thematic analysis was applied to six focus group interviews with 27 first responding personnel in Norway. The article describes the complexity and simultaneousness of the professional response. Three main themes were identified: (a) establishing contact, (b) diverse, supportive assistance, and (c) a complex helping context. The analysis showed that experiences from previous encounters and the deceased’s illicit drug use affected many of the professionals’ assessments, and implied an evaluation of the bereaved as not in need of emergency services or psychosocial follow-up. Professionals should be trained to understand drug-related death as a sudden and unnatural death, and to initiate immediate psychosocial crisis intervention. There is a need for further research on the perspective of professionals in the health and welfare services on the drivers and barriers to support (bereaved persons) during the acute phase.

## Abstract (es)

Studien har som mål å gi økt kunnskap om hvordan ansatte i kommunale helse-og velferdstjenester møter etterlatte i akuttfasen ved narkotikarelatert død. Refleksiv tematisk analyse ble brukt på seks fokusgruppeintervjuer med 27 akutthjelpere i Norge. Artikkelen beskriver kompleksiteten og samtidigheten i den profesjonelle responsen. Tre hovedtemaer ble identifisert: (a) etablering av kontakt, (b) variert, støttende bistand og (c) en kompleks hjelpekontekst. Analysen viser at erfaringer fra tidligere møter og de avdøde sin illegale rusbruk innvirket på mange av fagpersonene sine vurderinger, og førte til en vurdering av de etterlatte som ikke å være i behov for krisetjenester eller psykososial oppfølging. Fagpersoner bør opplæres til å forstå narkotikarelaterte dødsfall som brå og uventet død, og til å iverksette psykososial akuttberedskap. Det er behov for ytterligere forskning om profesjonelle i helse-og velferdstjenestenes perspektiv på hva som muliggjør og hindrer hjelp til etterlatte i den akutte fasen.

## Introduction

Drug-related mortality rates are on the rise. (Norwegian Institute of Public Health, 2020). Drug-related deaths (DRDs) comprise overdoses and fatalities related to mental or behavioral diseases and addiction (Amundsen, 2015). Due to its epidemic proportions, it is a public health concern. The Centers for Disease Control and Prevention report the highest numbers of death from overdose in the USA, with 70,630 fatal overdoses in 2019 compared to 5769 in the European Union (EU). The mortality rate for males aged 35–39 was more than double the average for all ages (as estimated by the European Monitoring Centre for Drugs and Drug Addiction in 2021). The mortality rate in Norway was 324 in 2020, a rate of 6.1/100 000, among the highest in Europe (Norwegian Institute of Public Health, 2020), with approximately 10–15 close family members/close friends left bereaved by every DRD (Dyregrov et al., 2020). The number of persons bereaved through overdose is significant: in Norway, there were at least 3240 such bereaved persons in 2020, and in the USA, there were between 706,300 and 1,059,450 such bereaved in 2019. Individuals bereaved through DRD have received little attention in the literature, although the sparse research that does exist shows a lack of support and understanding for the bereaved after a drug or alcohol-related death (Feigelman et al., 2012; McKell et al., 2018, p. 143; Richert et al., 2021; Templeton et al., 2017; Titlestad et al., 2020; Titlestad et al., 2021); Valentine, 2017; Valentine et al., 2016).

Losing a close family member/friend to a DRD can have consequences both for the individual and for society. Factors that may complicate the bereavement process include the strain of lifestyle risks associated with severe drug use prior to the death, a lack of social support, disenfranchised grief, and stigma before and after the death (Christiansen et al., 2020; Doka, 1999; Feigelman et al., 2011; Lambert et al., 2021; Løberg et al., 2019; Titlestad et al., 2021). In addition to personal suffering, there are also financial and societal ramifications arising from loss of health. Research shows that parents who suffer bereavement through DRD are at a greater risk of both mortality and adverse physical and mental health outcomes, including prolonged grief (Christiansen et al., 2020; Dyregrov et al., 2003; Guy & Holloway, 2007; Li et al., 2005; Stroebe et al., 2017). Because of the health risks associated with bereavement through DRD, it is essential that the support system provide the bereaved with support that meets their needs (Norwegian Directorate of Health, 2016, pp. 18–19). The Norwegian Directorate of Health recognizes the need to improve the services provided to close family members/friends and other bereaved persons (Norwegian Directorate of Health, 2019, pp. 14–15).

First responders who attend an emergency situation include those typically first on the scene: the police, ambulance personnel, the attending physician, nurses, and fire workers (Sharp et al., 2020, p. 18–20). Specially trained nurses, for example, in anesthesia, ambulance work, and mental health work are often present in the attending teams. Clergy and undertakers may also be present. In a DRD situation, overdose and crisis teams’ members may be the first to meet with the bereaved. This meeting plays an essential gatekeeper role in terms of further assistance. How these first responding personnel relates to, perceive, and categorize the bereaved will have a crucial impact on how the services relate to the DRD-bereaved and how the bereaved perceive this assistance.

Norwegian municipalities are obligated to provide comprehensive and coordinated assistance to the bereaved in the event of a sudden and unexpected death (Norwegian Directorate of Health, 2016, pp. 18–19). Psychosocial follow-up during the acute phase (1–4 weeks after the event) must be based upon an evaluation of needs and circumstances and include an assessment of the service currently being provided. Crisis assistance and grief intervention services are provided on an individual basis to families or within a bereaved person’s social network (e.g., through psychoeducation services at school and in the workplace).

National guidelines recommend that municipalities arrange psychosocial follow-up through crisis teams in the event of a crisis. However, as municipalities are given latitude in the management of their services, there is significant variation in the organization and administrative structure of these services (Norwegian Directorate of Health, 2016, p. 19), and they may vary from one municipality to the next. Nevertheless, it is necessary to specify what the local routines are, for example, how to alert and cooperate with the crisis teams, which play a key role in psychosocial follow-up (Norwegian Directorate of Health, 2016, p. 19). The crisis guidelines emphasize taking a proactive approach, meaning that a crisis team member should actively seek to make contact with the affected person at an early stage and repeat this contact if it is initially rejected (Norwegian Directorate of Health, 2016, p. 14). The services provided throughout the country by crisis teams vary insofar as how they are organized, for example, whether they are outreach services and how long the follow-up lasts (from a week to a year) (Norwegian Directorate of Health, 2016).

The research on the services provided to DRD-bereaved persons is scarce. In particular, we know little about professionals’ experience of dealing with persons bereaved through DRD. The majority of the research has sought to understand how to appropriately serve persons bereaved through DRD from the bereaved’s perspective (Lambert et al., 2021; Templeton et al., 2016; Titlestad et al., 2019; Valentine et al., 2018; Walter et al., 2017).

Improved knowledge of professional helpers’ experience of dealing, during the acute phase of bereavement, with persons bereaved through DRD has great potential for providing insight into practices, barriers, and opportunities in this respect (Dyregrov, 2001; Norwegian Directorate of Health, 2016, pp. 60–61). In order to be useful, however, these insights must be closely linked to the institutional, cultural, and political context. One way of addressing the knowledge gap in this area is to establish knowledge from critical stakeholders’ perspectives. The first responding professionals are important stakeholders, and details about how they perceive and assess the needs during the acute phase of bereavement of persons bereaved through DRD may shed light on possible gaps between the experience of such bereaved persons and the understanding and capability to provide adequate services of the organization concerned. The various context-sensitive insights of first responding professionals may be crucial to the process of developing better training. This study aims to conduct a rigorous qualitative exploration of the perspectives of first-responding health and welfare professionals on how they relate to meeting during the acute phase of bereavement with persons bereaved through DRD in Norway. The research question that we explore is: how do municipal health and welfare professionals relate to persons bereaved through DRD during the acute phase?

## Method

### Context of the study

This article is part of a larger project on drug death bereavement, “the END project” (“Etterlatte ved Narkotikarelatert Død”), initiated at Western Norway University of Applied Sciences in 2017. The main aim of the END project is to improve the life situation of the bereaved following a DRD.

### Study design

This article presents analyses derived from focus group interviews and demographic data collected in a questionnaire. Because there have been few studies on the assistance provided to people bereaved through DRD (McKell et al., 2018; Titlestad et al., 2019), we have taken a flexible, inductive, and empirically driven approach in order to find out about the experience of the professional helpers who provide such assistance. To this end, we employed purposeful sampling and reflexive thematic analysis (Braun & Clarke, 2019b), as interaction among participants in focus groups can promote synergy and spontaneity, and participants can comment on, explain, share, and discuss their opinions and experiences (Malterud, 2012, p. 18; Willig, 2013, pp. 30–31).

### Recruitment, sample, and participants

The recruitment of professional helpers to participate in focus group interviews was begun in the spring of 2019. Target municipalities in different locations were identified and contacted in conjunction with the Norwegian Directorate of Health’s pilot project for Norwegian municipalities with a high incidence of overdose deaths. The END project manager informed the leaders of the crisis teams orally and in writing of the criteria for the composition of the focus groups. In order to recruit professional helpers who represent diverse organizations and demographics (i.e., helpers who come from all parts of the country, including smaller and larger communities, as well as being diverse in terms of gender, education, and occupational position), the crisis team leaders were asked to identify relevant professional helpers in their municipality in accordance with the selection criteria and form focus groups in collaboration with the interview teams from the END project. The criterion for recruitment was that the professional helpers be in a position where they meet with bereaved persons after a DRD through the various municipalities’ health and welfare services and NGOs. They were asked to refer to their experience to shed light on the psychosocial follow-up for the bereaved after a DRD and to explore opportunities for cooperation and improved service along with others in the focus group interviews. All potential participants were given an informed consent form informing them that participation was fully voluntary and consisted of one focus group interview of approximately 2.5 hours’ duration and the completion of a questionnaire.

A total sample of 105 professional helpers was recruited from six target municipalities. This sample was organized into four main groups of participants: (1) first responding and emergency personnel who were likely to be first on the scene, (2) professionals from the municipal services who generally meet with bereaved persons, (3) representatives of various NGOs offering services to bereaved populations, and (4) the heads of various municipal services, such as emergency and outpatient rooms and mental health and addiction services. The participants in the four groups represented municipal psychosocial crisis teams, the police, emergency medical services, non-governmental organizations (NGOs), clergy, undertakers, and health and welfare services. Altogether, 24 focus group interviews were conducted; that is, there was one group for each of the four main groups in all six target municipalities.

The sample for the current study consists of the 27 professional helpers from group one; that is, the first responding professional helpers from the acute phase (Table 1). These 27 participants were organized into six groups: four groups of five participants, one group of six participants, and one group of one (i.e., an interview of one participant). Each group consisted of participants with different vocational backgrounds.

**Table 1.** Characteristics of focus group interviewees (N = 27).

Agency/Service	Qty	Specific information
Police	3	One of these individuals is also a member of a crisis team
Ambulance	3	
Overdose Team	3	One team member is located at a supervised injection site and another is at a low-threshold service
Crisis Team	12	Three of these individuals are with child welfare and one of them is also a priest. The rest are located in the various health, social, and addiction service districts
Emergency Care Center	2	
Priest	3	One of the priests is also a crisis team member and is accounted for there; two of the priests are located in hospitals
Undertaker	1	

There were twice as many females (*n* = 18) as males (*n* = 9), a typical distribution among such professional helpers.

Fifteen percent of the participants were 30–39 years of age, 37% were 40–49 years of age, 30% were 50–59 years of age, and 18% were 60 years of age or older. Two-thirds (67%) of the participants had completed their education more than 15 years prior to the interview. When asked how many bereaved persons they had met during the previous year (2018), 26% answered none or that they did not know, 48% and 15% reported 1 to 5 and 6 to 10 bereaved persons, respectively, and 11% had met with 11–29 bereaved persons. One of the participants had lost a loved one as the result of a DRD.

### Data collection

The focus group interviews were conducted during the fall of 2019 and the spring of 2020. Interviews were conducted in person, either at or near the participants’ workplace. Before an interview began, the participants completed a questionnaire on an e-pad or on paper. Some of the questions related to the participants’ background (e.g., age, education and time in position), and some questions quantified the approaches to help take in the municipalities of the individual helpers. The interviews were led by three teams, each consisting of a moderator—an experienced senior scientist—and an assistant (the co-leader). The senior scientists who acted as moderators were experienced in the conduct of focus group interviews. The moderators and co-leaders had a two-hour preparatory meeting led by the END project leader to discuss leveraging the benefits of the focus group method and securing the structure and atmosphere so as to co-create the best possible data. The members of the interview teams practiced their roles and use of the technical equipment so as to present themselves in a confident and assuring manner. There was a procedure guide to ensure that the conduct of all interviews was consistent with the information provided to the group, that is, in terms of interview structure and management. A theme guide ensured that the focus groups focused on the same key themes. A primary objective was to create a balance between structure and openness to generate more ideas, yield more profound insights into the problem under investigation, and manage the dynamics and interaction among group members (Morgan, 2010; Tausch & Menold, 2016).

The interview guide addressed the following: (1) the experience of community services in assisting the bereaved after a DRD, (2) the critical conditions and barriers connected with helping the bereaved, (3) professional helpers’ thoughts regarding the help needed by the bereaved after a DRD, (4) professional helpers’ views on opportunities for cooperation with and help from municipalities, social networks, voluntary organizations, and users, (5) forms of cooperation between public agencies, and (6) the use of key guides and management documents.

On average, the focus group interviews lasted between 1.5 and 2.5 hours, which was within the suggested time frame. The shortest interviews involved the fewest participants.

The interviews were audiotaped and transcribed verbatim by a professional transcriber. In addition, the interviewers immediately summarized their impressions in writing after the interviews. The first responder/acute phase transcripts consist of 174 pages of text.

## Analysis

Initially, the first author read all of the data several times to familiarize herself with the content and to form an overall impression. She noted her initial thoughts about possible themes, coding schemes, and meaning units for the data set as a whole and put these into a table. The coding process then proceeded over multiple re-readings, and an initial list of codes began to develop. The first author and one of the co-authors read, discussed, and re-read the content of the table and the meaning units and began identifying possible themes on the basis of the initial codes. Then the other two co-authors read and discussed the coding units and main themes, and all of the authors discussed the findings, reaching a consensus was reached and generating three main themes (see “Results” section).

Reflexive thematic analysis was applied to the interview data (Braun & Clarke, 2019b). Reflexivity can be defined as “continual self-awareness and critical self-reflection by the researcher on his or her assumptions, biases, predispositions, and actions, and their impact on the research situation and evolving interpretations” (Johnson & Christensen, 2019, p. 299). In an inductive approach such as this, the researcher does not engage with theoretical literature in the first stages. An inductive bottom-up approach was taken in which themes were searched for throughout the data set in order to identify repeated patterns of meaning through a coding process that was strongly linked to the data (Clarke et al., 2015). As this was an iterative process, the analysis and coding coincided in time. Braun and Clarke (2019a) emphasize the researchers’ contribution to this reflective process. Accordingly, the researchers discussed how their professional background and experience might interfere with the interpretative processes in their analyses. They also discussed factors that might potentially influence the interview process, such as differences among the interview teams. The authors’ occupational backgrounds include psychiatric nurse with an MA, psychologist with a Ph.D., social worker with a Ph.D., and sociologist with a Ph.D. They have long-term professional experience in health and welfare services and in research on crisis and traumatic grief, addiction, and mental health. The authors’ backgrounds are significant in understanding the dynamics and utterances in the group interviews and the interpretation during the analytical process.

Descriptive analyses (frequencies) were performed in SPSS of the background data of the participants (see Table 1).

## Ethical considerations

Approvals from the Norwegian Regional Committees for Medical and Health Research Ethics (reference number 2017/2486), the Norwegian Centre for Research Data (reference number 525,501), and Western Norway University of Applied Sciences were obtained prior to the sub-studies commencing. All research and dissemination follow the Helsinki Declaration to ensure the highest level of ethical research standards, including participant anonymity and confidentiality. According to the Norwegian Personal Data Act, the data has been managed in accordance with the General Data Protection Regulation, the instructions from the Norwegian Regional Committees for Medical and Health Research Ethics, and the regulations of Western Norway University of Applied Sciences regulations, and it is securely stored on a server at this University.

## Results

**Figure 1.**
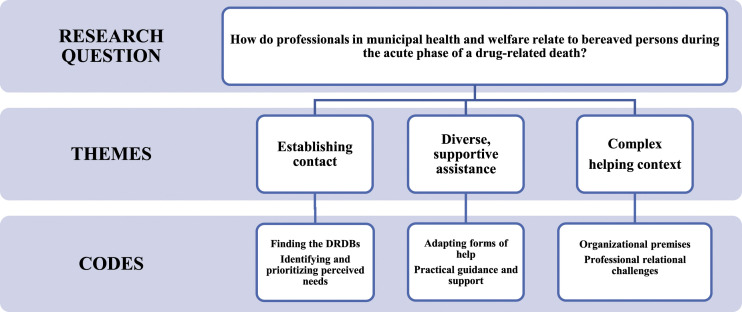
Health and welfare professionals’ relationships to drug death bereaved in the acute phase.

Three interconnected main themes were identified: (I) establishing contact, (II) diverse and supportive assistance, and (III) complex helping context (Figure 1). Codes for these themes are provided below and quotations have been selected to illustrate them.

### I. Establishing contact

In Norway, a municipal emergency communications center alerts the police and ambulance services, which rush to the address. The communications center, which is most often the municipality’s contact point, receives information from the first responders at the scene and subsequently connects the appropriate services. We found that the proactive support system that is in place to locate those bereaved after a DRD affects the transition to professional help during and directly after the acute phase. We also found that professional discretion was involved in many of the processes involved in locating the correct bereaved person. We coded these findings as (a) finding the DRD-bereaved and (b) identifying and prioritizing perceived needs.

#### a) Finding the DRD-bereaved

Although there may be formal procedures for notification, our material shows that actual praxis is contextual. It is not always apparent who is considered a bereaved person and subsequently notified of a death. Our material shows that, for the most part, close friends or family within the drug-using community are seldom categorized as being bereaved through unexpected death. A mental health and drug-related service leader who is also a member of the local crisis team problematized the difficulty of finding persons bereaved through DRD due to the perceived taboo of the topic. At the place of death, the actors involved behaved differently towards the bereaved that were present on the basis of their immediate evaluation of the actual situation. Proactive behavior can lead to a rapid response, but it can also lead to a lack of clarity in the emergency communications center in respect of who to notify or not notify. For example, some police officers would report directly to a crisis team if they felt that a situation was traumatic for the bereaved who were present. Some crisis teams would contact the emergency communications center to inquire whether the bereaved had been offered assistance. These crisis teams had become aware of a DRD as a result of the proximity of their premises. A crisis team member in a small town who already had a professional relationship with a now bereaved person contacted the police and agreed to perform the notification and the eventual follow-up together with a colleague. Participants from crisis teams needed to ensure that the bereaved knew that help was available, even if the bereaved rejected their initial attempt to make contact. The latter could be a dilemma, leading to discussions about the extent of proactivity, “whether it is phone terror,” and when to let go.

It could be challenging to locate relatives with the legal right to be informed of the death. A local crisis team member problematized the complexity in (ethical) praxis and legislation in this citation about finding the right bereaved (i.e., to attend solely to those legally defined as family members):

[W]e have ended up a bit in conflict lately about the notification because the legislation says something about it being the wife and children that you are obliged to notify. So, I do not know if the legislation has really fully followed the development of family structures in recent years. Anyhow, we ended up in a number of conflicts where people are not notified because this is not debated, but we had several who died where no one knew if he was married. And then it may happen for example that a wife is notified, and she notifies no one else as there isn’t any contact (in the family), so someone has to … call the siblings and parents. Anyway, this we have experienced … the somewhat ethical…. Are we allowed to call and notify (other family members)? Do we need to clarify first? (Group 3, ID13)

Bereaved persons who were concurrent drug users might already be well known to first responding services. Nevertheless, it could be challenging to determine who the significant others were during a phase when the timing is critical. The nature of a drug-related lifestyle (e.g., heavy intoxication, impulsive behavior, and unstable housing) may contrast with the crisis services’ organizational structures and usual service lines when the latter are trying to locate the bereaved, give notice, or provide immediate follow-up. In many of the focus groups, the participants discussed how to reach these multifaceted individuals, as the focus group participants were well informed of the increased risk of overdose in the time shortly after such an incident. Adding to the complexity, bereaved individuals who were concurrently using drugs might be intoxicated when given notice.

#### b) Identifying and prioritizing perceived needs

The various occupational and personal backgrounds of the different actors had an impact on how they understood and interpreted the bereaved person’s condition. The individuality of each context, situation, person, and relation was emphasized in all of the focus group interviews. We found that the perceived psychosocial needs of bereaved persons present at the place of death might be neglected due to the professionals’ priorities. This was presented as a dilemma for the ambulance workers or police officers in our focus groups. For instance, the ambulance workers focused on saving lives. If unsuccessful, they had to attend to the dead body in collaboration with the police present. If possible, ambulance personnel or police would remain with the distressed bereaved until other professionals (i.e., from the crisis team) arrived at the scene. However, if the police determined that a criminal investigation was necessary, forensic measures would have to be taken into account, which both affected and limited the ways in which other first responders could support the bereaved. In such a case, the bereaved might not be allowed near the dead person at the scene, or they might have to follow the police to the police station for questioning. This was viewed as an often challenging relational situation for our police participants.

A crisis team and municipal health service member illustrated the importance of assessing the immediate needs of the next of kin when meeting the bereaved. This is not solely a matter of immediate psychosocial follow-up; it is also necessary to consider the mobilization of possible individual resources. This is often a silent assessment based on experience and professional skills, and it may be essential both to the further healing process and to the evaluation and choices that are made in the steps that follow:

Well, there is something about pulling them out of the paralysis and grief that they are experiencing and starting to do something. I think most people benefit from that. So there is a very delicate balance in being their supervisor, their helper, but not taking over. It is vital that you mobilize people’s own resources that you see that they possess, but which may not be quite present because they are so paralyzed by grief and what they have experienced. It is essential to mobilize their own resources quickly. (Group 5, ID22)

The professional in this quote emphasized the importance of activating the bereaved person’s resilience even at the earliest stage. Although in the moment there may be nothing to indicate the bereaved person’s self-healing resources, these experienced professionals drew on their knowledge of recovery from shock and crisis psychology. They evaluated the here and now while also taking a long-range perspective on the best way to help.

### II. Diverse, supportive assistance

Acute psychosocial follow-up after an unnatural death involves the normalization and stabilization of reactions, information and psychoeducation, and economic and social relations. Practical support was assessed as an important part of helping the bereaved deal with the chaos and incomprehensibility during the acute phase. The focus group discussions illustrated how the bereaved are individuals with a variety of relationships, experiences, and expectations in respect of the system. Occasionally, a professional helper or bereaved person’s previous negative experience could have a bearing on how an offer of support was perceived and on its acceptance or rejection. Crisis team members reported encountering the anger and resentment of bereaved family members who had unsuccessfully sought help when their relative or close friend was alive.

#### a) Adapting forms of help

This code comprises issues related to conveying the message of death, existential care (receiving, containing, and processing immediate reactions), stabilization, and normalization. Information about the course of events in the days to follow was given and repeated in the interviews. Below, a clergyman illustrates several aspects of professional consideration as this relates to adapting information to meet the needs of the individual bereaved person and elaborates on the significance of the latter being informed of all kinds of specific details:

But anyhow, my concern is that they have someone around, and that there is someone there in the services that they can contact, or that they are in a system, without me entering into and organizing their whole life … but I must support them and make sure that they have the safety that they need. It is my concern that relatives get the information that they need and to put them in contact with the police and others so that they can find out more about what has happened. This is about working a bit structurally … to integrate such knowledge into the mourning process, and preferably into the support they will need further on. (Group 4, ID20)

#### b) Practical guidance and support

This code demonstrates the diversity of professional support available so as to best meet the needs of the bereaved during the acute phase, when they were often confused and bewildered. Practical involvement was regarded as important and was incorporated into immediate follow-up. Crisis team members would provide information to the other services involved, drive the bereaved to and from emergency care, and take them to the hospital for identification purposes or to the mortuary to see the dead person for the last time. If asked, members of the crisis team also attended the funeral. Undertakers provided guidance and advice to help the bereaved make good choices for the long term. Crisis teams with outreach services offered professional advice to the wider community, especially in cases that affected children and adolescents, for example, providing information and brochures to teachers, reaching out to daycare facilities, providing class assistance, and helping out with grief and memorial services. Experienced crisis team members addressed concerns about the deceased’s finances and helped make contact with the probate court to ensure that relatives did not inherit drug-related debt.

### III. Complex helping context

The assessment of bereaved persons’ needs in situations of unnatural and sudden death is complex. Matters such as organizational compartmentalization, juridical issues, and psychosocial reactions and relations are affected. At the first meeting, the professional helpers constantly assessed and balanced the bereaved person’s needs on the basis of their perception of requirements in that particular situation, all contingent upon what was as yet unknown, the “IF.” The professional background of the helpers and their position in the first responding services affected their evaluation of the situation. DRD seemed to add additional challenges, which affected how the professional helpers understood and performed their services. The majority of these professionals emphasized the importance of a gentle and compassionate approach, regardless of whether the bereaved reacts with anger, screams, tears, or hysterical laughter to what often appears to be a completely unexpected death. They stressed that these are natural reactions to painful events. We coded complex situation-related help responses according to two codes: (a) organizational premises and (b) professional relational challenges.

#### a) Organizational premises

Sudden unnatural death is usually handled by psychosocial follow-up services. As our interviews made clear, this is not the case when dealing with a DRD. The majority of our participants said they had not thought of persons bereaved through DRD as being within the scope of psychosocial follow-up services prior to being invited to participate in this study. Participants from one municipality stated that there had been no DRDs. Such understandings were significant in terms of how our participants classified the group of DRD bereaved organizationally as being within acute psychosocial help services or within a drug-related service, which subsequently affected the support for the bereaved:

Because, if we have the word “sudden death”, then, in a way, we are in our sudden death package of measures and not in our drug abuse package of measures. And that triggers, among other things, one thinking, oh yes, we should call upon the crisis team in the home municipality. Otherwise, you would have called NAV (Norwegian Labor and Welfare Administration) in the home municipality, which might not have thought of crisis teams because they have integrated the same stigma as what we have, right. So, by referring to it as sudden death, then you are on your way, then you have your foot in other types of help measures. Whether this actually would mean something to the bereaved, I do not know, but I think that for us, it is difficult to remember the bereaved in an overdose death because, in a way, the loved one's drug use has been the focus for so long. (Group 4, ID21)

Crisis team members’ primary position and role might be in other departments in the municipality. We found that the organizational position of a crisis team could have consequences for reaching bereaved persons in need of immediate crisis assistance. If a bereaved person had previously encountered the same professional, for example, a child welfare service worker who had assessed custody, this encounter could significantly affect how the bereaved person regarded the offer of assistance:

For us, it can be problematic that we are in child welfare and that they have met us in a different role, or that they know that crisis teams also deal with child welfare and that they may have difficulty trusting that we can differentiate the roles. … [W]e are very concerned about doing that, and it is crucial that people be confident in our providing psychosocial support. But it can be challenging, and … it can be very chaotic. In families where there have been dysfunctionalities, with conflicts between family members and a lot of long-term problems, we have to take this into consideration and challenge ourselves a little bit more. (Group 3, ID15)

#### b) Professional relational challenges

Several participants admitted to not reflecting much on how living with a close family member who was using illicit drugs might affect the bereaved person’s reactions during the acute phase. This code encompasses contributing factors anchored in the professional’s personal skills, sensibility, and discretion, for example, identifying opportunity structures exceeding habitual procedures and understandings. Under this code, the professional helpers’ capacity to be aware of their emotional reactions, vulnerabilities, and relational challenges is regarded as part of the ongoing communication process in their professional meetings with the bereaved. In some situations, these professionals’ capacities are even more challenged, as this low-threshold service worker shared:

I have experienced young people dying of an overdose, knowing that the mother has done it… I think there is something demanding about being a helper. We have many people left bereaved after drug-related death who have huge problems due to the very upbringing they have had in a highly dysfunctional family. … I think this has been very demanding on me, and I have had a lot of supervision in this regard because I can get so angry…. Sometimes, we just want to get the relatives out of the way (before death) because we think they are such a great burden, causing so much pain, and then it is difficult to contact them again after they have lost their family member. So I think that this also affects how well we manage to work with the bereaved sometimes. (Group 6, ID26)

In this quote, the crisis service worker’s professional empathy was challenged due to the lifestyle consequences of drug addiction passing on to the next generation. Hence, this professional helper’s attitudes may have a negative effect on their professional relationships. Some professional helpers were well aware of their vulnerability when personal values were challenged and sought professional counseling. In one of the focus groups, a few participants kept returning to the challenges of “dysfunctional families” without relating to or problematizing their personal attitudes in the given situation.

Another circumstance that affected how first responder personnel related to those bereaved through DRD was the intoxication of the bereaved. In this situation, the first responder’s need to look after their own safety while also interacting with the intoxicated person, and being expected to offer a compassionate presence can be both a dilemma and a stressor. Many of the participants emphasized that individuals bereaved through DRD should not and would not be approached differently from those bereaved through other types of sudden death. Nevertheless, some crisis team members reported that, when dealing with known drug users, follow-up had to take place in their service’s office because this was assessed as a more volatile situation. For the safety of the professional helpers, these bereaved persons were not offered home visits.

Then we have to go a few rounds with thoughts on how to do this follow-up, in terms of both safety for ourselves and the reactions of the bereaved. I think it can be different when they are under the influence of drugs than if they, in a way, have a pure grief reaction … reactions may include most things I think, and unlike many of us, they are acting out more if they are influenced by drugs. (Group 3, ID13)

## Discussion

As part of Norway’s social obligations, first responding professionals are expected to encounter and consider all kinds of situations while also taking account of ethics and legislation (Molander & Terum, 2008). However, individuals bereaved through DRD report a lack of support. Most DRD-bereaved persons encounter a complex service system that is difficult to navigate, professional helpers who lack knowledge, and, subsequently, challenging communication (Feigelman et al., 2011; Lambert et al., 2021; Richert et al., 2021; Titlestad et al., 2019; Valentine et al., 2018). Our material finds that the complexity of first responding services, the number and variety of professionals and their particular focus, and the need to react urgently may give rise to best practice challenges. This is in line with the existing research literature (Brataas, 2021; Dyregrov et al., 2000). Relating to a DRD-bereaved person during the acute phase is a complex process with a variety of occasions when it is possible to miss out on meeting the bereaved’s need for services. Psychosocial follow-up during the acute phase comprises miscellaneous tasks in response to crisis and shock reactions, existential care, practical help, and societal help, and this follow-up occurs across a range of organizational structures. The need for these responses to occur simultaneously affected how the first responding helpers related to and adapted their services.

### Locating the bereaved

There are multiple first responder services that deal with those bereaved through unexpected and unnatural death and they must react quickly. To give notice to the bereaved means finding the right persons if they are not present at the place of death. Locating the bereaved and assessing whether they need help and subsequently offering an appropriate service is a seemingly simple task on paper but in practice it proves to be complex with various occasions when it is possible to miss out on identifying a bereaved person in need of assistance. As found in other studies of unnatural death (Dyregrov, 2008), timing seemed critical to the professional helpers in this study. One example is the importance of giving devastating news quickly, respectfully, and compassionately, so that the bereaved does not receive this information from other sources such as social media or the press. In Norway, the police are legally obliged to notify the next of kin. This task is often delegated to others, such as local clergy (who may or may not alert the crisis team), or given directly to the crisis team (Regjeringen.no, 2002). In the capital, some districts have outsourced this responsibility to a security service with round-the-clock accessibility. A dilemma with this might be a random security guard not asking the essential questions that a trained health professional would ask.

### Organizational premises

In Norway, the municipality is responsible for providing coordinated medical and psychosocial services related to potentially traumatizing events (MHCS, 2018).

Assessing whether the bereaved or other persons involved need crisis assistance involves multiple organizational, cultural, professional, situational, and individual processes. It is, therefore, a process that is vulnerable to overlooking DRD-bereaved persons. We found that organizational premises could impede the provision of professional services for persons bereaved through DRD during the acute phase. Our analysis showed that psychosocial follow-up is linked to organizational premises, including the procedures for communication and actual practice from the scene of the death until the establishment of therapeutic relations with the bereaved persons who are in need of services. At the same time, national guidelines give municipalities the freedom to organize their services within their local context if this is justifiable (Norwegian Directorate of Health, 2016, pp. 21–25). This is consistent with our findings. In order to provide flexible and adapted professional assistance, it is possible to organize a seamless transition between divisions or administrative levels, and this was done on many occasions. Formal by-laws on organizational structures, limitations, and divisions were ignored on the basis of previously established productive relations in respect of the bereaved. For instance, a professional helper who provided the death notice would also provide follow-up and consider further mental help service without any formalities. This is an example of how the guidelines’ flexibility accommodates and optimizes seamless services. At the same time, however, this may create confusion as to who is involved and what has been stated, possibly giving rise to missing out on providing services to the bereaved (Norwegian Directorate of Health, 2016, pp. 18–19).

Economic or other resources are usually of decisive importance to the organizational structure of crisis teams (Norwegian Directorate of Health, 2016, p. 19). Our analysis illustrates how the organizational flexibility discussed above has led professionals to have multiple, sometimes conflicting, positions (e.g., police officers and welfare service workers). The professional helpers indicated that contact with the bereaved prior to the death, especially in situations where the helpers had been obliged to use force, such as where child welfare was involved, has affected how the services assessed and organized the help provided after the death. Our findings show how, in a situation characterized by the unexpected nature of sudden death, both helpers and the bereaved are affected and confused by these multiple roles. For example, if the police suspect criminal activity, they must attend to forensic procedures that prevail over adopting a caring approach at the scene.

### The immediate response is mirrored by the type of death

An important finding of our study is that, on occasion, the professionals’ individual understanding of DRD also affects how service providers relate to the DRD-bereaved, indicating that not only organizational premises but also personal attitudes towards illicit drug use can be a contributing and perhaps overshadowing factor in how some helpers relate to this group of bereaved persons (Doka, 1999, pp. 37–39; Thornicroft et al., 2007). There are several challenges involved in assessing the needs of the bereaved after a sudden and unnatural death, perhaps even more so in a DRD context. Professional discretion plays a part in the individual assessment of further needs, but it could be a potential source of missing out on providing services to bereaved persons who need assistance. Discretion is influenced by contextuality and performativity, for example, the services that these professionals are affiliated with, their knowledge of the bereaved person’s legal rights, and relational and crisis competence (Molander, 2016, p. 20). Collaboration with further services could thus be affected by; for example, various service sections localized understanding of the professional secrecy limitation.

How professional helpers classify the bereaved affects what further help is offered even during the acute phase. In an unexpected death situation, these helpers would usually have crisis follow-up in mind. Our findings suggest that this may be less certain when the cause of death is drug-induced, when the way of thinking about psychosocial follow-up may change. For example, the discourse on criminality versus a health and care perspective (Lambert et al., 2021; Titlestad et al., 2019; Valentine, 2017) suggests that many drug-death-bereaved persons find that the assistance they receive is affected by the cause of death. Some of the crisis team members in the focus group interviews associated the bereaved with the drug use of the deceased and determined that drug-related services would provide the best assistance in the situation. Drug-related considerations seemed to supersede crisis procedures in our data and to activate a chain of services different than those that would otherwise be allocated to suddenly bereaved persons. This may be perceived as professional helpers expressing spillover stigma related to the deceased person’s illicit drug use (Goffman, 2018; Van Boekel et al., 2013). For instance, we found that some of our participants seldom thought of drug death as a sudden, unnatural, and potentially traumatizing death. Nevertheless, as many of these professionals emphasized, a person bereaved through DRD should be treated the same way as any other suddenly bereaved person. To this end, it seems essential that first responding personnel understands that DRD can, in fact, be perceived as unexpected and potentially traumatizing for the bereaved and should thus be included in municipal crisis services (Barry et al., 2014; Bielenberg, 2018; McKell et al., 2018; McNeil, 2021; Nowak, 2015; Richert et al., 2021).

### The immediate response is influenced by demanding relationships

Professional mental health workers are trained to assess their reactions and not to act upon their own bodily or emotional impulses (Schibbye, 1995, pp. 29–44; 2009, pp. 57–100, 243–335). Our crisis team members observed, registered, suppressed, and reflected upon their own reactions to the bereaved person’s reactions. According to our findings, psychosocial follow-up during the acute phase entails performing heterogeneous tasks in response to the crisis, reactions of shock, and the need for existential care and practical and social assistance. The findings indicate extremely complex considerations and evaluations at different intra- and interconnected levels that challenge these professionals’ capacity to analyze, mentalize, and reflect. As stated in the expertise literature, a variety of reactions and expressions that are characteristic of shock and crisis are also characteristic of an unexpected and unnatural death situation (Brataas, 2021; Dyregrov et al., 2000; Kristensen et al., 2012; Norwegian Directorate of Health, 2016). First responders may thus be exposed to the intense emotional and physical reactions of the bereaved (Brataas, 2021, pp. 212–214; Norwegian Directorate of Health, 2016, p. 48). Bodily and emotionally paralyzed and numb, the bereaved may be incapable of maintaining their psychological self, as their personality structure may temporarily collapse (Brataas, 2021; Stevenson, 2017, pp. 1–54). In a situation like this, a professional approach offers a “holding environment,” as described by Winnicott (1965). The professional helper is more or less physically contouring the person in shock, framing the person when everything is perceived as fluid and incomprehensible. This is an organic process, where the professional evaluates the effect of supportive interventions on the bereaved person’s reactions and makes adjustments accordingly. The findings suggest there is interaction on a highly professional level. The complexity of assessments and the constant shifting between multiple levels and foci create both personal and professional challenges and a risk of missing out on critical considerations in terms of immediate psychosocial follow-up. Small mishaps or misjudgments may lead to misunderstandings and further destabilization of the situation. Relational components may suffer and thus contribute to what the bereaved view as a lack of understanding and compassion (Feigelman et al., 2011; Lambert et al., 2021; Richert et al., 2021; Titlestad et al., 2019).

## Strengths and limitations

This study’s strength is that it is part of a larger study which is a source of extensive qualitative data material and which ensures methodological stringency. The choice of focus groups resulted in a productive convenience sample and enhanced reflective processes among the participants because they were able to relate to each other and make the most of the processes for reflexive thematic analysis (Braun & Clarke, 2019b). We found that the way the participants related to persons bereaved through DRD was affected when those participants were unaware of some of the public services in their own municipality. In many of the focus groups, participants were surprised by their lack of an overview of existing services and said that they would have acted differently had they known that certain services existed.

We have sought to clearly describe the recruitment procedure, and we have made the analytic process explicit in the descriptions of both the method and the findings. Validity and transparency have been improved by way of numerous quotations, which allow the reader to assess the suitability of the themes. Moreover, the members of the interview teams were paired so that an experienced senior researcher conducted the interviews and discussed and reflected on the process with the other researcher, a process which has been described by the first author. Finally, the analytical trustworthiness is considered good, as all of the authors discussed the data and findings together.

Weaknesses relate to the general challenges of focus group methodology. There was no guarantee that the participants were those with the richest experience in their respective municipalities. All of our data has been derived from the participants’ experience, lack of experience, assumptions, and interpretations. Some of them had met each other before as colleagues, whereas others had not. At a minimum, previous encounters affected the start of the group discussions. We also discussed possible bias and confounders in our choice of municipalities with regard to demographics, geography, and exposure to those bereaved through DRD, for instance, how to group the various professionals and their dynamics and interaction flow, and how best to facilitate communication processes during the interviews.

A finding that arose from our cross-sectional grouping of first responding participants led to an enhancement of existing knowledge about an organization and available services. Consequently, cross-over connections were made. This is an excellent example of how the focus group methodology can create new knowledge as a result of sharing and reflecting together (Braun & Clarke, 2013; Malterud, 2012).

## Conclusion

This study set out to examine how municipal health and welfare services relate during the acute phase to persons bereaved through drug-related deaths. This is a complex process in which nurses, among multiple professional services, provide expert, cross-organizational individual, and social care and where there are various occasions on which these services may miss out on addressing the bereaved’s need for services.

The number and variety of professionals, their particular focus, and the need to take urgent action may lead to challenges in terms of best practice.

We found that, in many cases, first responder municipal health and welfare workers do not include persons bereaved through DRD in crisis services/psychosocial follow-up. This suggests that the association with illicit drug use and previous encounters affect the way that many professionals relate to the bereaved. A helpful approach in emergency situations might be for professionals to be trained first and foremost to acknowledge that DRD is unnatural and sudden death and to initiate immediate crisis intervention for the bereaved. Organizational premises, proactivity, and professional relations are central to these services and should be carefully attended to. We suggest that these services be integrated so that drug-related services offer individually adapted psychosocial follow-up for persons bereaved through DRD. This is already required in Norway’s current national guidelines. Further research is necessary to investigate what the municipal health and welfare services perceive as drivers and barriers during the acute phase and how they can optimize cooperation between the bereaved, the health and welfare services, and NGOs.
